# Examining the Influence of Maternal and Household‐Level Factors on the Global Diet Quality Score During Pregnancy in India

**DOI:** 10.1111/mcn.70221

**Published:** 2026-08-16

**Authors:** Shivani Kachwaha, Parul Christian, Andrew L. Thorne‐Lyman, Phuong Hong Nguyen

**Affiliations:** ^1^ Department of International Health Center for Human Nutrition, Johns Hopkins Bloomberg School of Public Health Baltimore Maryland USA; ^2^ Nutrition, Diets, and Health Unit International Food Policy Research Institute Hanoi Vietnam

**Keywords:** diet quality, India, influencing factors, maternal nutrition, pregnancy

## Abstract

Adequate nutrition during pregnancy is critical for mothers and newborns, but few studies have characterized maternal diets. The Global Diet Quality Score (GDQS) is a new metric to evaluate population diets. This study examined GDQS during pregnancy and its influencing factors in rural Uttar Pradesh, India. Data were from the Alive & Thrive (A&T) impact evaluation study, a cluster‐randomized trial testing behavior change communication delivered through the antenatal care (ANC) platform. We analyzed multi‐pass 24‐h dietary recalls combined from two cross‐sectional surveys, at baseline (2017, *n* = 660) prior to intervention and endline (2019, *n* = 674). The GDQS was constructed based on grams/day of 25 food groups consumed (range 0–49); for 16 healthy food groups (GDQS+), higher scores indicated higher consumption, whereas for 9 unhealthy food groups (GDQS*–*), scoring was reversed with higher scores reflecting lower consumption. Maternal and household factors influencing GDQS examined in a multivariable linear regression model included age, education, parity, nutrition knowledge, ANC visits, household size, religion, caste, socio‐economic status (SES), and food insecurity, adjusted for pregnancy duration, survey time, and A&T intervention. The mean (SD) at 18.5 (3.4) for overall GDQS, 7.3 (3.2) for GDQS+, and 11.2 (1.6) for GDQS‐ was low. Factors positively associated with overall GDQS and GDQS+ were 4+ ANC visits (*β* = 0.83 and 0.78), nutrition knowledge (*β* = 0.12 and 0.13), and SES (*β* = 0.82 and 0.96). Maternal education (*β* = 0.66) and caste (*β* = −0.66) were significantly associated with GDQS+ while no factors were associated with GDQS–. This study highlights poor diet quality during pregnancy in this context.

## Summary

- Adequate nutrition during pregnancy is critical for the health and nutritional outcomes of mothers and their newborns, but evidence on maternal diets is limited.
- This study from rural India highlights that dietary quality during pregnancy is low, characterized by low consumption of both healthy and unhealthy food groups.
- The findings emphasize that education and SES inequities persist in accessing a healthy diet whereas nutrition knowledge and recommended antenatal care may improve diet quality.
- Future research is recommended to validate the Global Diet Quality Score among pregnant women across diverse settings.

## 1 Introduction

Maternal dietary intake is a critical determinant of maternal and child nutrition status. Sub‐optimal nutrition during pregnancy leads to adverse outcomes, including small for gestational age babies, pre‐term births, low birth weight, increased maternal and newborn morbidity and mortality, and reduced cognitive ability in later life (Marshall et al. 2022; Victora et al. 2021). A recent analysis reported that 1.2 billion or 69% of women of reproductive age were deficient in at least one of three micronutrients (iron, zinc, vitamin A) (Stevens et al. 2022). During pregnancy, iron deficiency anemia was prevalent in 15‐20%, and vitamin A deficiency in 15% of pregnant women in LMICs (Gernand et al. 2016).

Population‐based studies across South Asia report a high prevalence of micronutrient deficiencies among pregnant women for zinc (15%–74%), vitamin B‐12 (19%–74%), and vitamin E (50%–70%) (Gernand et al. 2016). In India, recent National Family Health Survey data (NFHS) indicated that nearly 20% of women were underweight and 52% of pregnant women were anemic (International Institute for Population Sciences IIPS, & ICF 2021). Several nutrients have increased requirements during pregnancy, driven by fetal demands for growth as well as the metabolic and physiologic changes during pregnancy (Christian 2010; Vricella 2017).

A systematic review of 62 studies in LMICs revealed that maternal diets were characterized by imbalanced macronutrients (e.g., excessive consumption of carbohydrates and inadequate intake of protein and fat) and inadequate micronutrients due to predominantly cereal‐based diets (Lee et al. 2013). In India, data from 10 states reported a low median energy intake and about 35% and 20% of pregnant women had inadequate intakes of protein and fat, respectively (National Nutrition Monitoring Bureau 2012). The NFHS reported that less than 10% of pregnant women in India consumed meat, fish, or eggs, only 20% consumed fruits, and about half consumed pulses, dairy, and vegetables daily, with little improvement between 2006 and 2016 (International Institute for Population Sciences 2006, 2017). Evidence from sub‐national studies showed that minimum dietary diversity (MDD‐W, at least 5 out of 10 food groups) was low in Uttar Pradesh (25%–30%) and Bihar, and ~50% in Odisha and Chhattisgarh (Nguyen et al. 2021; Unisa et al. 2021).

Diet quality comprises aspects of nutrient adequacy (the intake of macronutrients and micronutrients in sufficient quantities), food variety or diversity (consuming a variety of food groups), and moderation (limiting the intake of unhealthy foods) (Trijsburg et al. 2019). The Global Diet Quality Score (GDQS) is a recently developed food‐based index to capture these multiple dimensions simultaneously, and has been validated among women of reproductive age (15–49 years) in diverse LMIC settings (Bromage et al. 2021). Unlike commonly used indicators such as the Minimum Dietary Diversity for Women (MDD‐W), which reflects only dietary diversity as a proxy for micronutrient adequacy, the GDQS includes a broader set of 25 food groups considering both healthy and unhealthy groups, and accounts for quantities consumed (Bromage et al. 2021). This allows GDQS to capture both nutrient adequacy and aspects of diet quality related to non‐communicable disease risk (Intake–Center for Dietary Assessment 2021). Evidence suggests GDQS performed similarly as the MDD‐W in capturing nutrient adequacy, while additionally being associated with lower odds of low mid‐upper arm circumference and anemia) (Bromage et al. 2021). The use of GDQS in pregnancy is limited, and there is a need to comprehensively understand diet quality among pregnant women in LMICs. While several socio‐demographic, pregnancy‐specific, and food environment factors have been identified to influence maternal diets, more evidence on the associations between different factors and diet quality, using the GDQS, is needed (Doyle et al. 2017; Nash et al. 2013).

Under the Alive & Thrive (A&T) initiative, the International Food Policy Research Institute (IFPRI) conducted a cluster‐randomized trial to evaluate the impact of a package of behavior change communication interventions on maternal nutrition in India (2017–2019). A&T aimed to strengthen the provision of maternal nutrition interventions (interpersonal counseling on diet diversity and quantity, iron folic acid and calcium supplements, and weight monitoring during pregnancy) within the existing government antenatal care (ANC) platform (Sanghvi et al. 2022). This paper describes the results of a secondary analysis using data collected as part of the A&T impact evaluation study to examine diet quality during pregnancy using the GDQS and to identify associated maternal and household‐level factors in India.

## 2 Methods

### 2.1 Alive and Thrive Intervention Study and Data Sources

The A&T impact evaluation study was designed as a two‐arm cluster‐randomized trial with repeated cross‐sectional surveys conducted at baseline (2017) and endline (2019). The surveys were conducted in 26 rural blocks across two districts (Unnao and Kanpur‐Dehat) in the state of Uttar Pradesh. The study population comprised pregnant women in the second or third trimester (*n* = 667 at baseline and *n* = 674 at endline) recruited through community‐based sampling. Details on the randomization procedure, sample size estimation, and data collection have been reported previously (Nguyen et al. 2020). Briefly, among the 26 rural blocks in Unnao and Kanpur‐Dehat, 13 were randomly assigned to intervention and 13 to control areas through manual lottery, using a pair‐matched randomization approach where blocks with a similar score on socio‐demographic characteristics were matched and treated as a pair. Within each block, 7 Gram Panchayats (village administrative units) were selected using probability proportional to size, with 1–2 villages and 300–350 households in each Gram Panchayat. A census of household listing was conducted before the survey to select four households with pregnant women in each Gram Panchayat using systematic random sampling (Nguyen et al. 2020). The A&T interventions did not change minimum diet diversity (MDD‐W consumption of ≥ 5 food groups in the last 24 h) or women's knowledge of adequate nutrition during pregnancy (Nguyen et al. 2021). The impact evaluation found that MDD‐W was higher at endline (45%) relative to baseline (35%) in both intervention and control areas with no significant differences between them.

The data sources relevant to this secondary analysis are summarized as follows. Dietary intake data was collected by trained interviewers using the multi‐pass, quantitative 24 h dietary recall method (Gibson and Ferguson 2008). This method includes four distinct steps or “passes” to ensure completeness and accuracy: (1) listing all foods or beverages consumed over the past 24 h; (2) collecting detailed information on each item consumed, recipe, and cooking method; (3) estimating quantities using standard measurement tools (e.g., utensils, scales); and (4) verifying the food list and including any forgotten items. For non‐standard recipes, the person with primary responsibility for preparing and distributing meals in the family was asked about recipes prepared, ingredients for these recipes, and the amount of these ingredients. Women were also asked about their micronutrient supplementation intakes and if yesterday was a special feasting or fasting day.

Information on household composition, socio‐economic status (SES), food insecurity, as well as women's knowledge on maternal nutrition and exposure to ANC services was collected from pregnant women at baseline and endline. Demographic data were collected using standardized questions on household caste, religion, and occupation. SES was measured using the questionnaire developed by the World Bank for LMICs (Gwatkin et al. 2007), which included several items on the house construction quality, asset ownership, and water and sanitation facilities. Food insecurity was assessed using the Household Food Insecurity Access Scale (HFIAS), a 9‐item questionnaire on different aspects of food insecurity experienced in the past month (Coates et al. 2007). Exposure to ANC was assessed by asking women whether they visited a health facility for ANC and the total number of visits during pregnancy. Maternal anthropometric data, including pre‐pregnancy weight and gestational weight gain, were not available in this cross‐sectional dataset.

### 2.2 Global Diet Quality Score

The GDQS was created for this analysis using the 24‐h dietary recall data at baseline and endline following prescribed guidelines (Intake–Center for Dietary Assessment 2021). The overall GDQS is a sum of points across 25 food groups, with a possible range of 0–49. The GDQS also includes 2 sub‐scores: the GDQS+ (range: 0–32) comprising the 16 healthy food groups and the GDQS–(range: 0–17) comprising the seven unhealthy food groups and the two food groups unhealthy in excess (Table S1) (Bromage et al. 2021).

Dietary data were processed to reflect the consumption in grams of each single food and ingredients of mixed recipes, which were then classified into the corresponding GDQS food group. Consumption levels based on grams consumed per day of each GDQS food group were defined as low, medium, and high; except for high‐fat dairy which had low, medium, high, and very high levels. Points were assigned per respondent and GDQS food group based on the consumption level in the past 24‐h period such that points associated with healthy food groups increased with higher consumption and points for unhealthy food groups decreased with higher consumption. Points for the 2 food groups unhealthy in excess (red meat and high‐fat dairy) increased to a specific amount and then decreased for higher consumption beyond that level. The GDQS variable (as well as the GDQS+ and GDQS–) were tabulated separately for baseline and endline as a population‐based metric by summing points across all 25 food groups and women.

### 2.3 Maternal and Household Factors

A conceptual framework presented in Figure S1 was developed for this study to guide decisions about including maternal and household factors in the dataset. The maternal‐level covariates for this analysis include age, education, parity, nutrition knowledge, and exposure to ANC. Maternal age was constructed as a continuous variable as age in completed years. Maternal education was constructed as a categorical variable based on years of schooling with 4 categories: no schooling, primary school, middle school, high school and above. Parity was categorized as nulliparous (0 previous live birth), 1 child, and 2 or more children. A nutrition knowledge score was created based on women's responses to questions assessing their knowledge of diet diversity and adequate quantity during pregnancy. For each question, a score of 1 was assigned if correct and 0 if not. A composite score was created by summing scores across questions and scaling to a 10‐point score. Exposure to ANC was constructed as a binary variable based on receipt of 4 or more ANC visits during pregnancy.

Household‐level covariates included household size, caste, religion, SES, and food insecurity. Household size was constructed as a continuous variable of the number of household members residing together. Caste was categorized as general caste, scheduled caste/tribe, and other backward classes. Religion was created as a binary variable as belonging to Hindu or other religions. A SES index was created from the first factor of the principal component analysis (PCA) as a composite measure of household construction quality, water and sanitation facilities, and asset ownership, and then divided into tertiles to reflect low, middle, and high levels of SES as a categorical variable (Vyas and Kumaranayake 2006). Finally, food insecurity was constructed as a categorical variable based on responses to the HFIAS questionnaire with four categories: food secure, mild insecure, moderate insecure, and severe insecure (Coates et al. 2007). All study covariates were tabulated separately for baseline and endline.

### 2.4 Statistical Analysis

Descriptive statistics were reported for all study variables as means and standard deviations (SD) for continuous variables and proportions and numbers for binary and categorical variables. Bivariate and multivariable linear regression models were employed to examine associations between maternal and household‐level factors and the overall GDQS, GDQS+, and GDQS–. Data from both study arms of the impact evaluation were combined in the analysis. The baseline and endline datasets were pooled to obtain a more robust sample size after examining results separately from summary statistics and estimates from bivariate and multivariable linear regressions analysis to verify comparability based on the means and direction and magnitude of coefficients. Among covariates, nutrition knowledge and ANC visits were time‐varying, but differences between baseline and endline were not influenced by the A&T intervention as noted above. Pregnancy duration in weeks, A&T intervention, survey time (baseline or endline), and the interaction of time and A&T were also included as covariates adjusted for in the model. Robust variances were used to adjust for the cluster design of the study at the block level using the cluster version of the Huber‐White robust estimator (Hayes and Moulton 2017), and the equation is described below.

GDQS(γ)=β0+β1age+β2i.education+β3i.parity+β4knowledge+β5i.ANC+β6hhsize+β7i.caste+β8i.religion+β9i.SES+β10i.foodinsecurity+β11pregnancyweek+β12A&T+β13time+β14A&Txtime

The coefficient associated with the interaction term, β14, shows the changes in GDQS between the A&T intervention arms and the baseline and endline survey periods. Model checking procedures included assessing multicollinearity using the Variance Inflation Factor. The Akaike Information Criterion was employed to check model fit and parsimony. Statistical significance was set at 5%, and all statistical tests were two‐sided. Beta coefficients and 95% confidence intervals were reported as regression analysis estimates. P‐values were reported as *p < 0.05, **p < 0.01, ***p < 0.001. All data analysis was conducted using Stata 17.0.

### 2.5 Ethics Statement

The study protocols, consent forms, and questionnaire were reviewed and approved by the Institutional Review Board at IFPRI in Washington, DC. Local approvals were also obtained in India from the Suraksha Independent Ethics Committee. All women were provided with detailed information about the study in writing and verbally at recruitment. Written informed consent was obtained from all women. The trial was registered at ClinicalTrials.gov (NCT03378141).

## 3 Results

### 3.1 Maternal and Household Sample Characteristics

Sample characteristics were similar at baseline and endline and combined descriptive statistics are reported as follows (Table 1). The mean (SD) age of pregnant women was 25 (4.1) years old. The mean (SD) pregnancy duration was 28.4 (6.6) weeks, and 35.2% and 64.8% of women were in the second and third trimesters of pregnancy, respectively. Among all women, 21.8% had no schooling, 15.6% had completed primary school, 22.2% secondary school, and 40.4% high school and above. In all, 38.5% of women were nulliparous, 27.8% had 1 child, and 33.7% had 2 or more children. The mean (SD) nutrition knowledge score (out of 10) was 4.3 (2). Only 10.6% of women reported having made ≥ 4 ANC visits during pregnancy. The mean (SD) household size was 5.4 (2.3) members and 92.1% belonged to Hindu religion. Among all households, 17.2% belonged to the general caste, 39.8% to the scheduled caste/tribes, and 43.1% to other backward classes. Most households (80.8%) were food secure based on the HFIAS, while ~6% each experienced mild, moderate, or severe food insecurity, respectively.

**Table 1:** Maternal and household characteristics.

	Total	Baseline	Endline
	*n* (%)/Mean (SD)	*n* (%)/Mean (SD)	*n* (%)/Mean (SD)
Women characteristics	*n* = 1341	*n* = 667	*n* = 674
Age, years	25.0 (4.1)	25.1 (4.1)	25.0 (4.0)
Duration of pregnancy, weeks	28.4 (6.6)	28.2 (6.1)	28.7 (7.1)
Pregnancy trimester			
Second trimester	472 (35.2)	243 (36.4)	229 (34.0)
Third trimester	869 (64.8)	424 (63.6)	445 (66.0)
Years of schooling	7.1 (4.6)*	6.9 (4.8)	7.4 (4.5)
Education category			
No schooling	289 (21.8)	161 (24.5)	128 (19.1)
Primary school	207 (15.6)	102 (15.6)	105 (15.7)
Secondary school	295 (22.2)	137 (20.9)	158 (23.6)
High school and above	536 (40.4)	256 (39.0)	280 (41.7)
Parity category			
0	516 (38.5)	245 (36.7)	271 (40.2)
1	373 (27.8)	202 (30.3)	171 (25.4)
≥ 2	452 (33.7)	220 (33.0)	232 (34.4)
Nutrition knowledge (0–10)^a^	4.3 (2.0)***	3.8 (1.9)	4.7 (2.0)
Number of ANC visits	1.8 (1.5)*	1.9 (1.6)	1.7 (1.4)
Early ANC (1st trimester)	518 (38.7)	266 (39.9)	252 (37.6)
4+ ANC visits	141 (10.6)	81 (12.2)	60 (8.9)
Household characteristics			
Household size	5.4 (2.3)**	5.2 (2.1)	5.6 (2.4)
Caste/class			
General	230 (17.2)	112 (16.8)	118 (17.5)
Scheduled caste/tribe	533 (39.8)	278 (41.7)	255 (37.8)
Other backward classes	578 (43.1)	277 (41.5)	301 (44.7)
Religion as Hindu	1235 (92.1)	614 (92.1)	621 (92.1)
Socio‐economic status			
Low	448 (33.4)	223 (33.4)	225 (33.4)
Middle	447 (33.3)	222 (33.3)	225 (33.4)
High	446 (33.3)	222 (32.3)	224 (33.2)
Household Food Insecurity^b^			
Food secure	1084 (80.8)	559 (83.8)	525 (77.9)
Mild insecure	82 (6.1)	39 (5.9)	43 (6.4)
Moderate insecure	84 (6.3)	48 (7.2)	36 (5.3)
Severe insecure	91 (6.8)	21 (3.2)	70 (10.4)

^a^ Composite score constructed by summing responses to questions on knowledge of diet diversity during pregnancy, ranging 0–10.

^b^ The Household Food Insecurity Access Scale (Coates et al. 2007) measures food insecurity experienced by households in the previous 30 days through 9 questions with the score ranging from 0 to 27 and categorized to reflect different levels of food insecurity. ANC: Antenatal care.

* *p* < 0.05

** *p* < 0.01

*** *p* < 0.001 report significant differences between baseline and endline; other differences non‐significant.

### 3.2 Global Diet Quality Scores Among Pregnant Women

**Figure 1:**
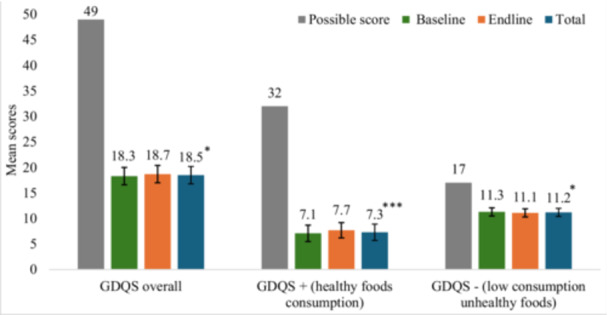
Global Diet Quality Scores among pregnant women. *Note:* Graph presents mean scores with standard deviations shown in error bars. *n* = 660 at baseline and 674 at endline. GDQS: Global Diet Quality Score. *p < 0.05, ***p < 0.001 report significant differences between baseline and endline.

**Figure 2:**
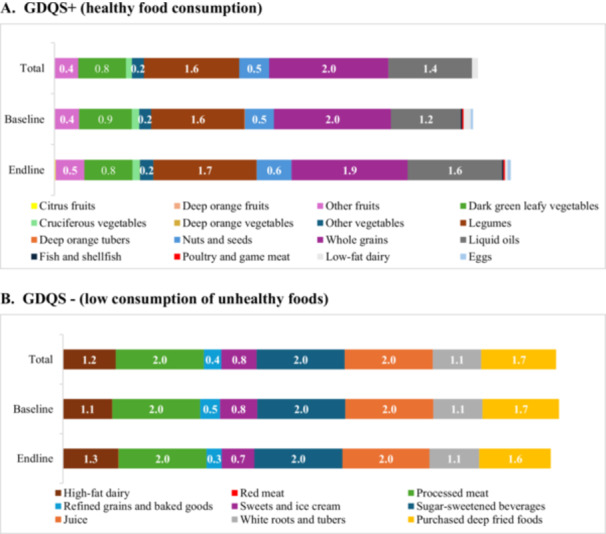
Mean point contribution of food groups to Global Diet Quality Score. A. GDQS+ (healthy food consumption). *Note:* Bar graphs represent the number of points contributed from each food group to mean baseline (7) and endline (7.7) GDQS healthy food consumption scores. *n* = 660 at baseline and 674 at endline. B. GDQS– (low consumption of unhealthy foods). *Note:* Bar graphs represent the number of points contributed from each food group to mean baseline (11.3) and endline (11.1) GDQS unhealthy food consumption scores. *n* = 660 at baseline and 674 at endline. GDQS, Global Diet Quality Score.

The overall GDQS and sub‐scores were similar at baseline and endline and combined descriptive statistics are reported. Out of a possible maximum total score of 49 points, the mean (SD) of overall GDQS was 18.5 (3.4) (Figure 1, Table S2). The GDQS+, which reflects consumption of the healthy food groups with a possible score of 32, had a mean (SD) of 7.3 (3.2). Reflecting low consumption of unhealthy foods, the mean (SD) of GDQS– was 11.2 (1.6) out of a possible score of 17 points. The key food groups that contributed the most points to the GDQS+ score at both baseline and endline included whole grains, legumes, liquid oils, dark green leafy vegetables, nuts and seeds, and other fruits whereas those contributing to the GDQS‐ included processed meat, sugar‐sweetened beverages, juice, and purchased deep fried foods (Figure 2, Table S3).

Most women (> 90%) consumed low amounts of citrus fruits, deep orange fruits/vegetables/tubers, fish and shellfish, poultry and game meat, low‐fat dairy, and eggs (Figure 3). Only 15%–25% of women had medium or high consumption categories for other fruits, dark green leafy vegetables, cruciferous vegetables, and nuts and seeds. Most women (60%–90%) had medium or high consumption categories for whole grains, legumes, liquid oils, and other vegetables. For the seven unhealthy food groups, all women (100%) had low consumption categories for processed meat, sugar sweetened beverages, and juice, and 80% had low consumption categories for purchased deep‐fried foods. A vast majority (70%–85%) of women had medium or high consumption categories of refined grains, sweets, and white roots and tubers. Finally, for the two food groups unhealthy in excess, most women (70%–80%) had medium or high consumption categories for high‐fat dairy, but only 3% had very high consumption, whereas all women had low consumption for red meat.

Figure 3Percent consuming low, middle, high categories of Global Diet Quality Score food groups. A. GDQS+ (healthy food consumption). *Note: n* = 1334 for combined baseline and endline. B. GDQS– (low consumption of unhealthy foods). *Note: n* = 1334 for combined baseline and endline. GDQS, Global Diet Quality Score.

**Figure:**
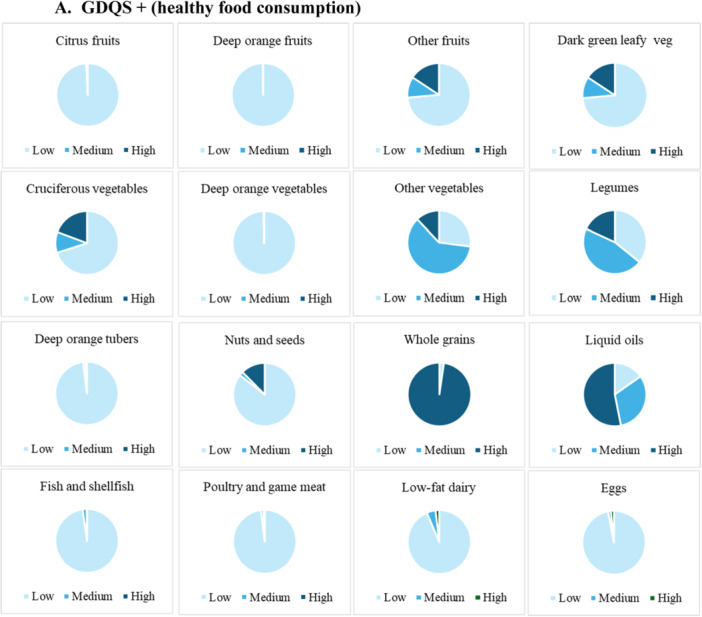

**Figure:**
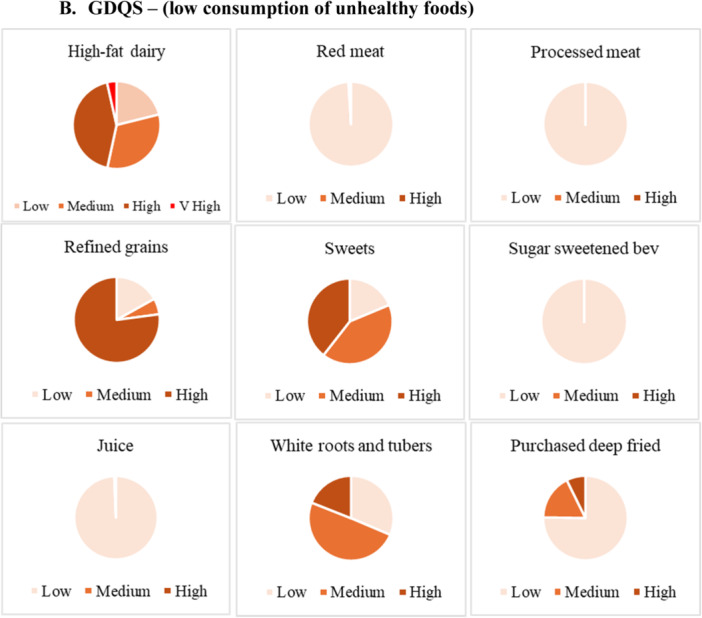

### 3.3 Maternal and Household Factors Influencing the Global Diet Quality Score

The bivariate associations of overall GDQS with maternal and household‐level covariates showed significant positive associations with maternal education (*β* = 1.25 for high school and above vs. no schooling), 4 + ANC (β = 1.18), nutrition knowledge (*β* = 0.18), and socio‐economic status (*β* = 1.47 for high vs. low SES) (Table 2). Significant negative associations were observed for parity (*β* = −0.88 for ≥ 2 vs. 0), caste (*β* = −1.08 for scheduled caste/tribes and *β* = −0.71 for other backward classes vs. general), and food insecurity (*β* = −0.76 for mild food insecurity vs. food secure). Positive associations remained significant in multivariable models for 4 + ANC visits (*β* = 0.83), nutrition knowledge (*β* = 0.12), and socio‐economic status (*β* = 0.82 for high vs. low SES).

**Table 2:** Bivariate and multivariable linear regression of maternal and household factors and the Global Diet Quality Score among pregnant women.

	Overall GDQS		GDQS+		GDQS–	
	Bivariate regression	Multivariable regression	Bivariate regression	Multivariable regression	Bivariate regression	Multivariable regression
	*n* = 1329	*n* = 1312	*n* = 1329	*n* = 1312	*n* = 1329	*n* = 1312
	*β* [95% CI]	*β* [95% CI]	*β* [95% CI]	*β* [95% CI]	*β* [95% CI]	*β* [95% CI]
Age (years)	−0.05* [−0.09, −0.00]	0.02 [−0.04, 0.08]	−0.04 [−0.08, 0.00]	0.02 [−0.04, 0.09]	−0.01 [−0.04, 0.02]	−0.01 [−0.04, 0.03]
Maternal education (ref: no schooling)						
Primary school	0.34 [−0.33, 1.01]	0.26 [−0.41, 0.93]	0.56 [−0.00, 1.13]	0.45 [−0.09, 0.99]	−0.22 [−0.60, 0.15]	−0.19 [−0.59, 0.21]
Secondary school	0.45 [−0.16, 1.05]	0.10 [−0.58, 0.78]	0.63* [0.08, 1.18]	0.27 [−0.23, 0.78]	−0.18 [−0.58, 0.21]	−0.18 [−0.62, 0.27]
High school and above	1.25*** [0.77, 1.73]	0.53 [−0.19, 1.24]	1.42*** [0.95, 1.89]	0.66* [0.09, 1.24]	−0.16 [−0.43, 0.10]	−0.13 [−0.51, 0.24]
Parity (ref: 0)						
1	−0.32 [−0.76, 0.12]	−0.19 [−0.64, 0.27]	−0.19 [−0.50, 0.13]	−0.04 [−0.39, 0.31]	−0.14 [−0.40, 0.13]	−0.15 [−0.42, 0.12]
≥ 2	−0.88** [−1.37, −0.39]	−0.49 [−1.17, 0.19]	−0.79*** [−1.15, −0.43]	−0.35 [−0.92, 0.23]	−0.09 [−0.34, 0.16]	−0.15 [−0.43, 0.13]
4+ANC visits	1.18** [0.45, 1.91]	0.83* [0.16, 1.49]	1.10** [0.45, 1.75]	0.78** [0.25, 1.32]	0.08 [−0.27, 0.42]	0.04 [−0.32, 0.40]
Nutrition knowledge score (range: 0‐10)	0.18*** [0.10, 0.27]	0.12* [0.02, 0.21]	0.21*** [0.13, 0.28]	0.13** [0.05, 0.21]	−0.02 [−0.07, 0.02]	−0.01 [−0.07, 0.04]
Household size	0.07 [−0.00, 0.14]	0.00 [−0.07, 0.08]	0.20 [−0.05, 0.09]	−0.05 [−0.13, 0.02]	0.05* [0.00, 0.09]	0.06* [0.01, 0.11]
Caste (ref: general)						
Scheduled caste/tribe	−1.08*** [−1.63, −0.53]	−0.50 [−1.10, 0.10]	−1.28*** [−1.75, −0.81]	−0.66* [−1.19, −0.13]	0.20 [−0.10, 0.50]	0.16 [−0.19, 0.52]
Other backward classes	−0.71* [−1.30, −0.12]	−0.43 [−1.02, 0.17]	−0.91*** [−1.38, −0.44]	−0.62* [−1.10, −0.13]	0.20 [−0.06, 0.46]	0.19 [−0.08, 0.46]
Hindu religion (ref: other)	−0.32 [−1.02, 0.38]	−0.33 [−1.02, 0.36]	−0.35 [−1.02, 0.32]	−0.41 [−1.08, 0.25]	0.03 [−0.28, 0.34]	0.09 [−0.24, 0.41]
Socio‐economic status (ref: low)						
Middle	0.40 [−0.02, 0.83]	0.18 [−0.31, 0.67]	0.40* [0.01, 0.79]	0.20 [−0.21, 0.62]	0.00 [−0.18, 0.19]	−0.02 [−0.25, 0.20]
High	1.47*** [1.00, 1.93]	0.82* [0.18, 1.46]	1.54*** [1.18, 0.79]	0.96** [0.42, 1.51]	−0.07 [−0.32, 0.17]	−0.14 [−0.48, 0.19]
Food insecurity (ref: food secure)						
Mild insecure	−0.76* [−1.52, −0.00]	−0.35 [−1.17, 0.47]	−0.71* [−1.39, −0.04]	−0.31 [−1.03, 0.40]	−0.05 [−0.33, 0.23]	−0.03 [−0.32, 0.26]
Moderate insecure	−0.35 [−1.25, 0.54]	0.15 [−0.73, 1.03]	−0.49 [−1.43, 0.46]	0.08 [−0.78, 0.95]	0.13 [−0.18, 0.44]	0.07 [−0.24, 0.38]
Severe insecure	−0.40 [−1.30, 0.50]	0.05 [−0.80, 0.91]	−0.52 [−1.46, 0.42]	−0.05 [−0.84, 0.75]	0.11 [−0.27, 0.50]	0.10 [−0.28, 0.47]
Pregnancy duration (weeks)	0.00 [−0.03, 0.03]	−0.01 [−0.03, 0.02]	−0.01 [−0.03, 0.02]	−0.02 [−0.04, 0.01]	0.01 [−0.00, 0.02]	0.01 [−0.00, 0.02]
Time (ref: baseline)	0.19 [−0.39, 0.78]	0.12 [−0.45, 0.69]	0.54 [−0.09, 1.17]	0.48 [−0.15, 1.12]	−0.37* [−0.74, −0.01]	−0.37*** [−0.56, −0.17]
Alive & Thrive (ref: control)	−0.31 [−0.91, 0.29]	−0.23 [−0.84, 0.37]	0.07 [−0.61, 0.74]	0.16 [−0.52, 0.84]	−0.35** [−0.54, −0.16]	−0.40* [−0.77, −0.03]
Time x Alive & Thrive	0.47 [−0.38, 1.32]	0.39 [−0.44, 1.23]	0.16 [−0.72, 1.04]	0.08 [−0.79, 0.95]	0.31* [0.02, 0.61]	0.31* [0.01, 0.62]

*Note:* Bivariate linear regression models of overall GDQS, GDQS + (healthy food consumption), and GDQS ‐ (low consumption of unhealthy foods) with each covariate, standard errors adjusted for clustering. Survey time is defined as 1 for endline and 0 for baseline. Multivariable linear regression models of factors associated with overall GDQS, GDQS + (healthy food consumption), and GDQS – (low consumption of unhealthy foods), adjusted for pregnancy duration, survey time (defined as 1 for endline and 0 for baseline), Alive & Thrive intervention, the interaction of time and intervention, and clustering.

Abbreviation: ANC, Antenatal care.

* *p* < 0.05

** *p* < 0.01

*** *p* < 0.001.

Similarly, for GDQS+, significant positive associations were observed for maternal education (*β* = 0.63 for primary school, *β* = 1.42 high school and above vs. no schooling), 4 + ANC (β = 1.10), nutrition knowledge (*β* = 0.21), and socio‐economic status (*β* = 0.4 for middle SES and *β* = 1.54 for high SES vs. low); while negative associations were observed for parity (*β* = −0.79 for ≥ 2 vs. 0), caste (*β* = −1.28 for scheduled caste/tribe and *β* = −0.91 for other backward classes vs. general), and food insecurity (*β* = −0.71 for mild food insecurity vs. food secure). In multivariable regression analysis, these associations remained for maternal education (*β* = 0.66 for high school and above), 4 + ANC (*β* = 0.78), nutrition knowledge (*β* = 0.13), and socio‐economic status (*β* = 0.96 for high SES), and caste (*β* = −0.66 for scheduled caste and *β* = −0.62 for other backward classes).

For GDQS–, estimates for most covariates were non‐significant in bivariate and multivariable regression analysis. Significant associations were observed for endline versus baseline (*β* = −0.37), A&T (*β* = −0.4), and time × A&T (*β* = 0.31) for GDQS– but not overall or GDQS+ (Figure S2). The multivariable linear regression analysis results for baseline and endline data separately showed largely similar findings in terms of the strength and direction of effect sizes for overall GDQS and sub‐scores. Associations of most maternal and household factors (specifically age, education, parity, ANC visits, nutrition knowledge, caste, and SES) with GDQS showed similar trends between baseline and endline, albeit with some differences in magnitude or statistical significance. These findings confirmed the decision to pool datasets along with consistent results from descriptive statistics across the two survey rounds (Tables S4 and S5).

## 4 Discussion

Our analysis of dietary data among pregnant women in rural Uttar Pradesh, India found diet quality was poor as characterized by an overall low GDQS, with low consumption of healthy foods but also low consumption of unhealthy foods. Among the 25 GDQS food groups, the healthy food groups that most contributed to the total score were moderate to high consumption of whole grains, legumes, other vegetables, and liquid oils, while the unhealthy food groups that mostly contributed to the GDQS– were high‐fat dairy and refined grains, sweets, white roots and tubers, but not red meat.

A comprehensive set of maternal and household‐level factors examined for their influence on diet quality revealed higher maternal education, SES, nutrition knowledge, and exposure to ANC to be independently and positively associated with higher GDQS whereas belonging to scheduled caste/tribes or other backward classes was associated with a lower GDQS. Poorer households from scheduled caste/tribes or backward classes in general had a lower GDQS, including low consumption of healthy food groups. Low consumption of animal source foods, including poultry, eggs, fish, and red meat, particularly may also be due to socio‐economic constraints and predominantly vegetarian dietary practices in this community (Bellows et al. 2020). These associations highlight the potential importance of access to critical ANC services which in addition to nutrition knowledge may alleviate poor diet quality in rural communities despite SES and related constraints.

Although statistically significant, the covariates contributed little to the overall GDQS variability; for e.g. there was a nearly 1‐point increase in the score for ANC and SES and around half a point or lower for household caste and nutrition knowledge. A 1‐point increase may translate to shifting from low to medium or medium to high consumption for some of the healthy food groups, linked to an additional consumption (for 1 group) of up to 50–90 g for fruits and vegetables, 30 g for eggs, and 100 g for dairy. Although related nutritional and health outcomes need to be assessed, these amounts are potentially of significance, especially in resource‐constrained environments. There was an attenuation observed in associations between GDQS and education, caste, food insecurity, and parity in the multivariable analysis, suggesting some shared explanatory variance, although high multicollinearity was not detected. Mean GDQS was higher at baseline in the control group and declined from baseline to endline resulting in a significant A&T intervention × time interaction. The relative worsening of the GDQS‐ was related to increased consumption of unhealthy foods in the control group, whereas there was no change in the intervention group.

Few previous studies in LMICs have examined GDQS in the pregnancy context. A study in rural Ethiopia examined GDQS among pregnant women and reported a mean score of 18.2, comparable with the results of this study (Kang et al. 2022). Another study in Northwest China reported a higher mean GDQS of 27.5 among pregnant women (Yang et al. 2022), while a study among pregnant women in Tanzania reported score as low as 2.4 (Cliffer et al. 2023). A study among adolescent girls in Vietnam showed similar results as this study for overall GDQS (17.9), GDQS+ (6.9), and GDQS– (11) (Nguyen et al. 2023). The GDQS validation study by Bromage et al. (2021) evaluated GQDS among women of reproductive age across 13 LMICs and reported a range of mean scores between 18 in Kenya—26.3 in Nigeria (Bromage et al. 2021; Bromage et al. 2021). Scores reported in this study were towards the lower end of this range. The validation study also established a cut‐off of GDQS < 15, indicating a poor quality of diet; however, this cut‐off has not yet been evaluated for pregnant women.

We identified important context‐specific and physiological considerations for the scoring of the unhealthy food groups in the construction of the GDQS. During pregnancy, increased requirements for nutrients such as iron, folate, and calcium may warrant higher rather than penalized consumption of some foods categorized as “unhealthy” in GDQS. Specifically, red meat and full‐fat unpasteurized milk or dairy products (more commonly consumed rather than skimmed or low‐fat) are an important source of protein and micronutrients in the Indian diet context which is generally less diverse and predominantly cereal based. Similarly, white roots and tubers (especially white potatoes) are a staple in rural LMIC contexts and important contributors to energy and nutrients, including vitamin C, potassium, and dietary fiber (King and Slavin 2013). Inclusion of these groups in the unhealthy category was a shortcoming of the GDQS in this context.

It is important to consider how the GDQS scoring of unhealthy foods groups translates to dietary practices in this setting. Given the low consumption of animal source foods, the negative scoring of red meat when consumed in high amounts had minimal influence on GDQS. In this population, ~75% of women consumed moderate or high amounts of high‐fat dairy, and 3% consumed very high amounts. A small proportion of women therefore received lower scores for excessive high‐fat dairy, although most women received higher scores for moderate‐high consumption. Negative scoring of white roots and tubers beyond around 100 grams (roughly 1 small white potato) likely impacted GDQS in this population, where 50% of women consumed moderate and 18% consumed high amounts of these foods based on GDQS consumption categories. It is worthwhile to evaluate and potentially refine consumption categories and cut‐offs for these food groups generally and also for pregnant women, given the increased dietary requirements during pregnancy.

We found a few factors to be associated with GDQS‐, in part due to the nearly uniform low consumption of unhealthy foods in this population. Previous studies on the determinants of unhealthy food consumption in LMICs have highlighted other relevant factors related to the food environment, including market availability, cost, as well as socio‐cultural factors such as habits, tastes, preferences, and traditional diets (Karanja et al. 2022; Westbury et al. 2021). Further research is needed to examine these drivers in relation to GDQS– in other LMICs, especially in contexts where diets are homogenous. It should also be noted that previous qualitative studies have highlighted the importance of other potential factors (not examined in this study) influencing maternal dietary diversity in these contexts, including social norms, beliefs and self‐efficacy, food taboos, food prescriptions and proscriptions during pregnancy, and family support (Jhaveri et al. 2023; Nguyen et al. 2021).

The strengths of this study include data from a cluster‐randomized trial with a large sample size of pregnant women. Robust data collection methods included quantitative multi‐pass 24‐h recalls assessing dietary intakes and standardized questionnaires to obtain socio‐demographic information. The study limitations include cross‐sectional data which limits analysis of temporal relationships as well as causal inferences of the associations found, although this was not the primary study objective. The data collection methods relied on self‐reported information, which has potential for recall bias and social desirability bias. Comprehensive recurrent field training and quality assurance and control measures were employed to alleviate potential biases during data collection. Finally, the one‐time measure of dietary intake in the context of the GDQS guidelines and non‐availability of repeated recalls in this study limits inferences to usual intakes due to lack of information about within‐person variation. While mean population intakes are expected to be unbiased when GDQS is analyzed as a continuous variable, random within‐person errors may attenuate associations and decrease statistical power. However, given the relatively large sample size in this study, the impact on statistical power is likely limited, although some attenuation of effect estimates cannot be ruled out. In addition, a greater proportion of women would be classified as having GDQS < 15 due to random errors, thereby underestimating diet quality in this context (Thorne‐Lyman et al. 2013), although we did not categorize women in our analysis.

To our knowledge, this is the first South Asian study to examine GDQS and its influencing factors in the pregnancy context and contributes to the essential evidence needed on diet quality during pregnancy in undernourished LMIC settings. The results of this study emphasize the poor quality of diet during the critical life stage of pregnancy in India and the inequities that persist in accessing a healthy diet. Specific sub‐groups of women were identified to have poor diet quality, such as poverty, low caste and education, and lack of exposure to health services. Future research is recommended to validate the GDQS across diverse LMIC settings, considering potential adjustments to the definition of unhealthy foods, and to examine changes in diet quality over time among women during pregnancy.

## Author Contributions

S.K. and P.C. developed the analytic strategy for secondary data and conceptualized the paper, P.H.N. and S.K. designed and conducted the impact evaluation study, S.K. conducted the data analysis and drafted the manuscript, P.C. provided feedback and inputs to the statistical analysis, P.C., A.T.L., and P.H.N. critically reviewed and provided edits and inputs to the draft manuscript. S.K. made the final revisions; and all authors: read and approved the final manuscript.

## Conflicts of Interest

The authors declare no conflicts of interest.

## Supporting information

Supporting File.

## Data Availability

The data that support the findings of this study are openly available in Harvard/Dataverse at https://hdl.handle.net/10568/144611, reference number https://doi.org/10.7910/dvn/hf0f79. All datasets have been anonymized and are publicly available on the Harvard Dataverse repository (https://dataverse.harvard.edu/dataverse/IFPRI).
