# Supplementary material for: Examining the Influence of Maternal and Household‐Level Factors on the Global Diet Quality Score During Pregnancy in India
Source: Matern Child Nutr. 2026 Aug 16;22(3):e70221. doi: 10.1111/mcn.70221 (PMC13477746; doi:10.1111/mcn.70221)
Supplement: Supplementary file 1 [file MCN-22-e70221-s001.docx]

**Supplemental Table 1. Global Diet Quality Score food groups and scoring criteria**

| **Classification** | **Food group** | **Categories of consumed amounts (g/day)** | | | | **Points assigned** | | | |
| --- | --- | --- | --- | --- | --- | --- | --- | --- | --- |
|  |  | **Low** | **Middle** | **High** | **Very high** | **Low** | **Middle** | **High** | **Very high** |
| **Healthy** | Citrus fruits | <24 | 24–69 | >69 |  | 0 | 1 | 2 |  |
|  | Deep orange fruits | <25 | 25–123 | >123 |  | 0 | 1 | 2 |  |
|  | Other fruits | <27 | 27–107 | >107 |  | 0 | 1 | 2 |  |
|  | Dark green leafy vegetables | <13 | 13–37 | >37 |  | 0 | 2 | 4 |  |
|  | Cruciferous vegetables | <13 | 13–36 | >36 |  | 0 | 0.25 | 0.5 |  |
|  | Deep orange vegetables | <9 | 9–45 | >45 |  | 0 | 0.25 | 0.5 |  |
|  | Other vegetables | <23 | 23–114 | >114 |  | 0 | 0.25 | 0.5 |  |
|  | Legumes | <9 | 9–42 | >42 |  | 0 | 2 | 4 |  |
|  | Deep orange tubers | <12 | 12–63 | >63 |  | 0 | 0.25 | 0.5 |  |
|  | Nuts and seeds | <7 | 7–13 | >13 |  | 0 | 2 | 4 |  |
|  | Whole grains | <8 | 8–13 | >13 |  | 0 | 1 | 2 |  |
|  | Liquid oils | <2 | 2–7.5 | >7.5 |  | 0 | 1 | 2 |  |
|  | Fish and shellfish | <14 | 14–71 | >71 |  | 0 | 1 | 2 |  |
|  | Poultry and game meat | <16 | 16–44 | >44 |  | 0 | 1 | 2 |  |
|  | Low-fat dairy | <33 | 33–132 | >132 |  | 0 | 1 | 2 |  |
|  | Eggs | <6 | 6–32 | >32 |  | 0 | 1 | 2 |  |
| **Unhealthy in excessive amounts** | High-fat dairy | <35 | 35–142 | >142–734 | >734 | 0 | 1 | 2 | 0 |
|  | Red meat | <9 | 9–46 | >46 |  | 0 | 1 | 0 |  |
| **Unhealthy** | Processed meat | <9 | 9–30 | >30 |  | 2 | 1 | 0 |  |
|  | Refined grains and baked goods | <7 | 7–33 | >33 |  | 2 | 1 | 0 |  |
|  | Sweets and ice cream | <13 | 13–37 | >37 |  | 2 | 1 | 0 |  |
|  | Sugar-sweetened beverages | <57 | 57–180 | >180 |  | 2 | 1 | 0 |  |
|  | Juice | <36 | 36–144 | >144 |  | 2 | 1 | 0 |  |
|  | White roots and tubers | <27 | 27–107 | >107 |  | 2 | 1 | 0 |  |
|  | Purchased deep fried foods | <9 | 9–45 | >45 |  | 2 | 1 | 0 |  |

**Reference:** Intake–Center for Dietary Assessment. (2021). The Global Diet Quality Score: data collection options and tabulation guidelines.

Supplemental Figure 1. Conceptual framework on factors influencing diet quality

Note: Highlighted factors are examined in this study. ANC: Antenatal care.

Supplemental Table 2. Global Diet Quality Score sub-scores among pregnant women

|  | **Baseline** | **Endline** |
| --- | --- | --- |
|  | **n= 660** | **n= 674** |
|  | **Mean (SD)** | **Mean (SD)** |
| ***GDQS + (Healthy, 0-32*)** | 7.1 (3.2) | 7.7 (3.1)^***^ |
| ***GDQS – (Unhealthy, 0-17)*** | 11.3 (1.6) | 11.1 (1.7)^*^ |
| ***Overall GDQS (0-49)*** | 18.3 (3.4) | 18.7 (3.4)^*^ |
|  | **n (%)** | **n (%)** |
| ***GDQS <15 (High risk)*** | 98 (14.9) | 71 (10.5) |
| ***GDQS ≥15 and ≤23 (Moderate risk)*** | 499 (75.6) | 522 (77.5) |
| ***GDQS ≥23 (Low risk)*** | 63 (9.6) | 81 (12.0)^*^ |

GDQS: Global Diet Quality Score. *p<0.05, ***p<0.001 report significant differences between baseline and endline.

Supplemental Table 3. Mean consumed amounts of Global Diet Quality Score food groups among pregnant women

|  | **Baseline** | **Endline** |
| --- | --- | --- |
| **Average intake (g/day)** | **Mean (SD)** | **Mean (SD)** |
| ***GDQS + (healthy food consumption)*** | **n= 660** | **n= 674** |
| Citrus fruits | 0.4 (5.5) | 1.4 (14.6) |
| Deep orange fruits | 0.0 (1.2) | 0.3 (6.6) |
| Other fruits | 35.4 (75.2) | 45.8 (87.0)^*^ |
| Dark green leafy vegetables | 15.8 (34.8) | 16.2 (38.4) |
| Cruciferous vegetables | 17.1 (32.3) | 20.1 (45.7) |
| Deep orange vegetables | 0.0 (0.0) | 0.5 (5.2)^*^ |
| Other vegetables | 52.3 (49.6) | 63.1 (59.2)^***^ |
| Legumes | 22.6 (27.5) | 25.3 (29.2) |
| Deep orange tubers | 2.0 (14.1) | 0.6 (6.5)^*^ |
| Nuts and seeds | 5.9 (20.5) | 7.4 (21.3) |
| Whole grains | 217.4 (141.3) | 221.5 (136.4) |
| Liquid oils | 10.0 (12.3) | 12.9 (10.7)^***^ |
| Fish and shellfish | 0.9 (6.1) | 0.8 (7.1) |
| Poultry and game meat | 0.8 (6.9) | 1.0 (9.0) |
| Low-fat dairy | 12.2 (51.5) | 3.8 (20.7)^***^ |
| Eggs | 1.5 (11.6) | 1.7 (11.8) |
| ***GDQS – (low consumption unhealthy foods)*** |  |  |
| High-fat dairy | 194.2 (219.3) | 238.6 (252.3)^***^ |
| Red meat | 0.0 (0.7) | 1.1 (10.8)^*^ |
| Processed meat | 0.0 (0.0) | 0.0 (0.0) |
| Refined grains and baked goods | 153.1 (145.6) | 172.8 (156.8)^*^ |
| Sweets and ice cream | 36.1 (37.4) | 37.4 (30.3) |
| Sugar-sweetened beverages | 0.0 (0.0) | 0.0 (0.0) |
| Juice | 0.0 (0.0) | 2.9 (26.2)^**^ |
| White roots and tubers | 64.1 (62.9) | 64.0 (61.9) |
| Purchased deep fried foods | 8.5 (23.0) | 12.8 (29.1)^**^ |

GDQS: Global Diet Quality Score. *p<0.05, **p<0.01, ***p<0.001 report significant differences between baseline and endline; other differences non-significant.

**Supplemental Figure 1. Difference-in-differences plots of GDQS by survey time and intervention group**

Note: Lines represent adjusted predicted means from multivariable linear regression models including a time × intervention interaction, with standard errors clustered at the block level. Error bars indicate 95% confidence intervals. GDQS: Global Diet Quality Score.

Supplemental Table 4. Multivariable linear regression of maternal and household factors associated with Global Diet Quality Score among pregnant women at Baseline

|  | **Overall GDQS** | **GDQS +** | **GDQS –** |
| --- | --- | --- | --- |
|  | **n = 643** | **n = 643** | **n = 643** |
|  | **β [95% CI]** | **β [95% CI]** | **β [95% CI]** |
| Age (years) | 0.02 [-0.07,0.12] | 0.03 [-0.06,0.12] | -0.01 [-0.06,0.04] |
| Maternal education (ref: no schooling) |  |  |  |
| Primary school | **0.94^*^** [0.01,1.87] | **1.22^*^** [0.31,2.14] | -0.28 [-0.63,0.07] |
| Secondary school | 0.40 [-0.34,1.13] | **0.83^**^** [0.23,1.43] | -0.43 [-0.89,0.02] |
| High school and above | 0.58 [-0.24,1.40] | **1.05^*^** [0.23,1.86] | **-0.47^**^** [-0.78,-.16] |
| Parity (ref: 0) |  |  |  |
| 1 | -0.30 [-0.89,0.30] | -0.08 [-0.63,0.48] | -0.22 [-0.54,0.10] |
| ≥2 | -0.67 [-1.67,0.34] | -0.51 [-1.38,0.36] | -0.16 [-0.50,0.18] |
| 4+ ANC visits | 0.45 [-0.19,1.09] | 0.53 [-0.06,1.13] | -0.08 [-0.50,0.33] |
| Nutrition knowledge score (range: 0-10) | 0.12 [-0.04,0.27] | 0.13 [-0.01,0.26] | -0.01 [-0.07,0.05] |
| Household size | -0.02 [-0.16,0.12] | -0.05 [-0.18,0.08] | 0.03 [-0.04,0.11] |
| Caste (ref: general) |  |  |  |
| Scheduled caste/tribe | -0.04 [-1.04,0.95] | -0.18 [-0.97,0.62] | 0.13 [-0.34,0.61] |
| Other backward classes | -0.30 [-1.38,0.77] | -0.48 [-1.34,0.37] | 0.18 [-0.24,0.60] |
| Hindu religion (ref: other) | -0.84 [-1.91,0.24] | **-1.51^*^** [-2.68,-0.33] | **0.67^**^** [0.24,1.10] |
| Socio-economic status (ref: low) |  |  |  |
| Middle | -0.12 [-0.89,0.64] | -0.09 [-0.76,0.58] | -0.03 [-0.32,0.25] |
| High | 0.78 [-0.10,1.66] | 0.68 [-0.13,1.49] | 0.10 [-0.30,0.49] |
| Food insecurity (ref: food secure) |  |  |  |
| Mild insecure | -0.56 [-1.65,0.52] | -0.50 [-1.54,0.53] | -0.06 [-0.45,0.34] |
| Moderate insecure | -0.35 [-1.50,0.79] | -0.32 [-1.37,0.72] | -0.03 [-0.43,0.37] |
| Severe insecure | -0.10 [-1.24,1.03] | -0.37 [-1.55,0.80] | 0.27 [-0.33,0.86] |
| Pregnancy duration (weeks) | 0.01 [-0.05,0.06] | -0.01 [-0.06,0.03] | 0.02 [-0.00,0.04] |
| Alive & Thrive (ref: control) | -0.26 [-0.87,0.35] | 0.12 [-0.56,0.81] | **-0.39^*^** [-0.76,-0.01] |

Note: Multivariable linear regression models of factors associated with overall GDQS, GDQS + (healthy food consumption), and GDQS – (low consumption of unhealthy foods), adjusted for pregnancy duration, Alive & Thrive intervention, and clustering. ANC: Antenatal care.

*p<0.05, **p<0.01, ***p<0.001.

Supplemental Table 5. Multivariable linear regression of maternal and household factors associated with Global Diet Quality Score among pregnant women at Endline

|  | **Overall GDQS** | **GDQS +** | **GDQS –** |
| --- | --- | --- | --- |
|  | **n = 669** | **n = 669** | **n = 669** |
|  | **β [95% CI]** | **β [95% CI]** | **β [95% CI]** |
| Age (years) | 0.01 [-0.06,0.07] | 0.02 [-0.05,0.09] | -0.01 [-0.05,0.03] |
| Maternal education (ref: no schooling) |  |  |  |
| Primary school | -0.44 [-1.48,0.60] | -0.30 [-1.08,0.47] | -0.13 [-0.76,0.50] |
| Secondary school | -0.17 [-1.20,0.85] | -0.29 [-1.16,0.58] | 0.12 [-0.45,0.68] |
| High school and above | 0.44 [-0.67,1.55] | 0.24 [-0.65,1.13] | 0.20 [-0.39,0.80] |
| Parity (ref: 0) |  |  |  |
| 1 | -0.06 [-0.69,0.56] | 0.05 [-0.47,0.57] | -0.11 [-0.47,0.24] |
| ≥2 | -0.37 [-1.20,0.46] | -0.27 [-0.93,0.39] | -0.10 [-0.54,0.33] |
| 4+ ANC visits | **1.37^*^** [0.24,2.51] | **1.15^*^** [0.22,2.08] | 0.22 [-0.32,0.76] |
| Nutrition knowledge score (range: 0-10) | **0.10^*^** [0.01,0.19] | **0.12^**^** [0.03,0.21] | -0.02 [-0.09,0.04] |
| Household size | 0.02 [-0.08,0.12] | -0.06 [-0.15,0.04] | **0.08^*^** [0.00,0.15] |
| Caste (ref: general) |  |  |  |
| Scheduled caste/tribe | **-0.84^*^** [-1.57,-0.10] | **-1.02^*^** [-1.81,-0.23] | 0.18 [-0.23,0.59] |
| Other backward classes | -0.43 [-1.31,0.46] | -0.61 [-1.37,0.15] | 0.18 [-0.18,0.55] |
| Hindu religion (ref: other) | 0.05 [-0.99,1.09] | 0.49 [-0.45,1.44] | -0.45 [-0.91,0.02] |
| Socio-economic status (ref: low) |  |  |  |
| Middle | 0.47 [-0.21,1.16] | 0.51 [-0.16,1.18] | -0.04 [-0.34,0.26] |
| High | 0.85 [-0.00,1.71] | **1.26^**^** [0.51,2.01] | -0.41[-0.91,0.10] |
| Food insecurity (ref: food secure) |  |  |  |
| Mild insecure | -0.19 [-1.37,0.98] | -0.22 [-1.26,0.83] | 0.02 [-0.36,0.40] |
| Moderate insecure | 0.63 [-0.59,1.85] | 0.41 [-0.88,1.70] | 0.22 [-0.45,0.89] |
| Severe insecure | 0.21 [-0.75,1.17] | 0.13 [-0.73,0.98] | 0.09 [-0.35,0.52] |
| Pregnancy duration (weeks) | -0.02 [-0.06,0.02] | -0.02 [-0.05,0.01] | -0.00 [-0.02,0.01] |
| Alive & Thrive (ref: control) | 0.14 [-0.56,0.85] | 0.20 [-0.40,0.81] | -0.06 [-0.43,0.31] |

Note: Multivariable linear regression models of factors associated with overall GDQS, GDQS + (healthy food consumption), and GDQS – (low consumption of unhealthy foods), adjusted for pregnancy duration, Alive & Thrive intervention, and clustering. ANC: Antenatal care.

*p<0.05, **p<0.01, ***p<0.001.
